# Comprehensive analysis of ICD-related lncRNAs in predicting risk stratification, clinical prognosis and immune response for breast cancer

**DOI:** 10.18632/aging.205002

**Published:** 2023-09-09

**Authors:** Yuli Wang, Tao Yue, Qingqing He

**Affiliations:** 1 Department of Clinical Laboratory, The Second Hospital, Cheeloo College of Medicine, Shandong University, Jinan, China; 2 Department of Thyroid and Breast Surgery, The 960th Hospital of the Chinese People’s Liberation Army, Jinan, China

**Keywords:** ICD-related lncRNAs, BRCA, risk signature, immune infiltration, prognosis

## Abstract

Background: Breast cancer (BRCA) represents a significant threat with high mortality rates due to relapse, metastasis, and chemotherapy resistance. As a regulated cell death process characterized by the induction of immunogenic signals, immunogenic cell death (ICD) has been identified as an effective anti-tumorigenesis approach. However, the comprehensive study and its clinical value of ICD-related lncRNAs in BRCA is still missing.

Methods: The transcriptome matrix and corresponding clinical information of BRCA patients were obtained from The Cancer Genome Atlas (TCGA) database. Pearson correlation analysis was performed to identify ICD-related lncRNAs (ICDRLs). To determine the prognostic value of the identified ICDRLs, univariate Cox regression analysis, LASSO algorithm, and multivariate Cox regression analysis were employed to construct a risk model. The prognostic risk model was subsequently evaluated using univariate and multivariate Cox regression analysis, as well as Nomogram analysis. *In vitro* experiments were also conducted to validate the bioinformatics findings using quantitative real-time PCR (qRT-PCR).

Results: We established a prognostic risk signature consisting of five ICDRLs. The prognostic value of this model was subsequently confirmed in guiding BRCA prognostic stratification. Furthermore, we explored the correlation of the risk score with various clinical characteristics and chemotherapy response. qRT-PCR result confirmed the abnormal expression of ICDRLs, which was consistent with the bioinformatics data.

Conclusions: Our findings provide evidence of the critical role of ICDRLs in BRCA and offer a novel perspective for exploring precise treatment options for BRCA patients.

## INTRODUCTION

In 2021, breast cancer (BRCA) surpassed lung cancer to become the most newly diagnosed cancer type in the world [1]. Combined with the fact of its high mortality, BRCA has become one of the most threatening malignant tumors in women [1]. Although the etiology of BRCA is not fully understood, it has been reported that the occurrence of BRCA involves a variety of factors, such as genetics, environment or aging [2]. Tumor invasion, metastasis and distant spread are the most common causes of death in BRCA patients [3, 4]. In recent years, since the application and development of a variety of novel combined treatment strategies, the survival time of BRCA patients has been significantly prolonged. However, many BRCA patients develop relapse, metastasis and chemotherapy resistance after treatment, which ultimately leads to patient death [5, 6]. Therefore, further research on new potential therapeutic targets for BRCA has positive clinical significance.

Immunogenic cell death (ICD) is a regulated form of cell death characterized by the release of tumor-associated antigens (TAAs) and danger-associated molecular patterns (DAMPs) from dying cancer cells, including cell-surface exposure of calreticulin, extra cellular adenosine triphosphate (ATP), and high-mobility group box 1 (HMGB1) [7]. By producing reactive oxygen species (ROS), induced endoplasmic reticulum (ER) stress leads to the immunogenicity of tumor cells, which is the first step in the ICD signaling cascade [8]. In recent years, ICD induction has been revealed to be one of the most promising approaches for building long-term immunity against tumors [9]. In triple-negative BRCA (TNBC), immune checkpoint inhibitor (ICI) treatment is one of the most successful immunotherapies [10]. In response to ICI, progression-free survival (PFS) and overall survival (OS) can be significantly improved [11]. The occurrence of response to ICI in TNBC is related to certain biological pattern, including tumor-infiltrating lymphocytes, programmed death ligand 1 (PD-L1), and non-synonymous mutations [12]. In addition, preclinical models and clinical trials confirmed that induction of ICD sensitizes TNBC to ICIs treatment in this process [13, 14]. Previous studies also showed that chemo/ radiotherapy-elicited TNBC cell-derived HMGB1 enhances antitumor immunity induced by activated CD8^+^ T cells, revealing the importance of DAMP in immunotherapeutic treatment of TNBC [15]. Additionally, some ICD-targeted drugs have also shown the impact on BRCA treatment. Teniposide, a DNA topoisomerase II inhibitor, was reported to induce ICD through the cGAS/STING pathway and effectively inhibit tumor progression [16]. Sr-4835, a CDK12/13 specific inhibitor, triggers ICD and initiates T cell-dependent tumor elimination in BRCA [17]. This evidence preliminarily indicates that ICD biomarkers may serve as candidate prognostic factors for BRCA, which has potential for further comprehensive research and clinical application.

In this study, we first constructed a prognostic risk signature with five ICDRLs by using BRCA cohort. The reliability and sensitivity of the signature were further verified. We also explore the correlation of the risk score with various clinical characteristics, anticancer immune status, and chemotherapy response. qRT-PCR also showed significant ICDRL expression differences in BRCA cell line, which was in line with the bioinformatics results. The findings highlight the critical role of ICDRLs in BRCA and provide a novel perspective for exploring the metabolic mechanism and treatment of BRCA.

## MATERIALS AND METHODS

### Transcriptome matrix collection of BRCA

The transcriptome matrix and corresponding clinical information of patients diagnosed with BRCA (BRCA) were obtained from The Cancer Genome Atlas (TCGA) database, accessible at https://portal.gdc.cancer.gov/. To ensure data quality, samples with missing survival information or less than 0 days of survival time were excluded from the analysis. The final cohort comprised 1069 BRCA samples, which were included in this study. The gene matrix was extracted using Perl scripts. The Ensembl Human Genome Browser GRCh38.p13 database, available at http://asia.ensembl.org/index.html, was used for mRNA expression annotation. Clinical information was obtained using Perl scripts from the TCGA database. Subgroup analysis by gender and M stage was not conducted due to marked differences in sample sizes.

### Exploration of ICD-related lncRNAs

In this study, a total of 33 ICD-related genes were collected from a previously published article (Supplementary Table 1). The expression levels of ICD-related genes in BRCA (BRCA) were extracted using Perl scripts and the R package “limma”. Pearson correlation analysis was then conducted to identify ICDRLs using a threshold of |correlation coefficient| > 0.4 and *P*-value < 0.001 (r > 0.4, *P* < 0.001). A total of 184 ICDRLs were identified for further analysis (Supplementary Table 2).

### Prognostic signature development using ICD-related lncRNAs

Univariate Cox regression analysis was performed using the R package “survival” to identify prognostic ICDRLs for BRCA. The least absolute shrinkage and selection operator (LASSO) algorithm, implemented with the R package “glmnet”, was utilized to determine the critical variables of prognostic ICDRLs. A multivariate Cox regression analysis was then conducted to construct the risk model. The risk scores were calculated using the following formula: Risk Score = (−2.276 × expression of LINC02511) + (−0.751 × expression of AL451085.2) + (−1.649 × expression of AL133467.1) + (0.449 × expression of AC092718.4) + (−2.548 × expression of LINC01055). Subsequently, the BRCA patients were categorized into low- and high-risk groups based on their median risk scores. The R packages “survival” and “ggplot2” were used to estimate overall survival (OS) rates and investigate the separation pattern by principal component analysis (PCA). The BRCA samples were randomly divided into training and validation cohorts at a 1:1 ratio, and the risk scores of each sample were calculated accordingly [18].

### Independence evaluation and immune infiltration landscape estimation

In this study, we conducted univariate and multivariate Cox regression analyses using the R package “survival” to examine the independence of the factors. A nomogram model was then developed using the R package “rms” to predict the 1-, 3-, and 5-year survival probabilities of patients based on clinicopathological characteristics and risk score. The prognostic performance of the model was validated using time-dependent receiver operating characteristic (ROC) analysis with the R package “timeROC”. Additionally, we evaluated the stromal and immune cells, as well as the ESTIMATE score and tumor purity, using the ESTIMATE algorithm and the R package “estimate”. Finally, we used the “CIBERSORT R script v1.03” and the R package “CIBERSORT” to estimate the 22 types of immune cells in BRCA samples.

### Tumor mutational burden, immune response and drug sensitivity analysis

The tumor mutation data of BRCA samples were obtained from the TCGA database in “maf” format. Perl scripts were utilized to extract the mutation data from the raw dataset, while the creation of a waterfall diagram was accomplished using the “Maftools” package. The Immunophenoscore (IPS) outcome was acquired from the TCIA database, which is accessible at https://tcia.at/home. To extract immune checkpoint inhibitor (ICI) expressions from the TCGA matrix, the R package “limma” was employed, and the expressions were then log2-transformed (expression + 1). The Genomics of Drug Sensitivity in Cancer (GDSC) database was employed to assess drug sensitivity (IC50) using “pRRophetic” from R.

### Functional enrichment analysis

In this study, the R package “limma” was utilized to identify differential expression genes (DEGs). A screening threshold was set at |Fold Change| ≥ 2 and *P*-value < 0.05 to select the DEGs. Gene set variation analysis (GSVA) was then employed to calculate the KEGG terms of BRCA patients in both low- and high-risk groups. A significance level of *P* < 0.05 was considered to indicate statistically significant differences. To further analyze the DEGs, we performed pathway enrichment using Gene Ontology (GO) and Kyoto Encyclopedia of Genes and Genomes (KEGG) analysis through the use of the “clusterProfiler” R package.

### qRT-PCR analysis

The MCF-7 and MCF-10A cell lines were obtained from the American Type Culture Collection (ATCC) and cultured in DMEM (Dulbecco’s Modified Eagle Medium) supplemented with 10% Fetal Bovine Serum, 1% penicillin/streptomycin, and 2 mM L-glutamine in a humidified incubator at 37°C with 5% CO2. To extract mRNA, the cells were cultured in appropriate culture dishes until they reached 70–80% confluency. Total RNA was extracted using TRIzol reagent according to the manufacturer’s instructions. The quality and quantity of the RNA samples were assessed using a NanoDrop spectrophotometer and gel electrophoresis. mRNA was subsequently purified from the total RNA using poly-T oligo-attached magnetic beads and eluted in RNase-free water. The quality and quantity of the purified mRNA samples were assessed using an Agilent 2100 Bioanalyzer. The primer sequences for lncRNAs are shown in the Supplementary Table 3.

### Wound healing assay

Cells were seeded in 6 cm culture plates, and the monolayer cells were wounded by scratching with sterile plastic 200 μl micropipette tips and photographed using phase-contrast microscopy immediately and 48 hrs after wounding. The assays were independently performed in triplicate. The migration distance of each cell was measured after the photographs were converted to Photoshop files.

### Cell invasion and motility assay

Invasion of cells was measured in Matrigel (BD, Franklin Lakes, NJ, USA) -coated Transwell inserts (6.5 mm, Costar, Manassas, VA, USA) containing polycarbonate filters with 8 μm pores as detailed previously. The inserts were coated with 50 μl of 1 mg/ml Matrigel matrix according to the manufacturer’s recommendations. 2 × 10^5^ cells in 200 μl of serum-free medium were plated in the upper chamber, whereas 600 μl of medium with 10% fetal bovine serum were added to lower well. After 24 hr incubation, cells that migrated to the lower surface of the membrane were fixed in 4% paraformaldehyde and stained with 0.5% crystal violet. For each membrane, five random fields were counted at ×10 magnification. The mean cell number was calculated and data were presented as mean ± sd. from three independent experiments done in triplicate.

### Statistical analysis

All statistical analyses were conducted using R software (version 4.1.0) and Perl scripts. To investigate the correlation between risk score and drug sensitivity, Spearman-ranked correlation analysis was utilized with a significance level of *p* < 0.05 considered to indicate statistically significant differences. Differential functions between the two groups were analyzed using the Wilcoxon rank-sum test, and a significance level of *p* < 0.05 was considered to indicate statistical significance.

### Data availability statement

The datasets analyzed for this study are available in the Cancer Genome Atlas (TCGA) (http://tcga-data.nci.nih.gov/tcga/) and the Genotype-Tissue Expression Project (GTEx) (http://www.gtexportal.org/home/index.html) databases.

## RESULTS

### Identification of prognostic-associated ICDRLs in BRCA

**Figure 1:**
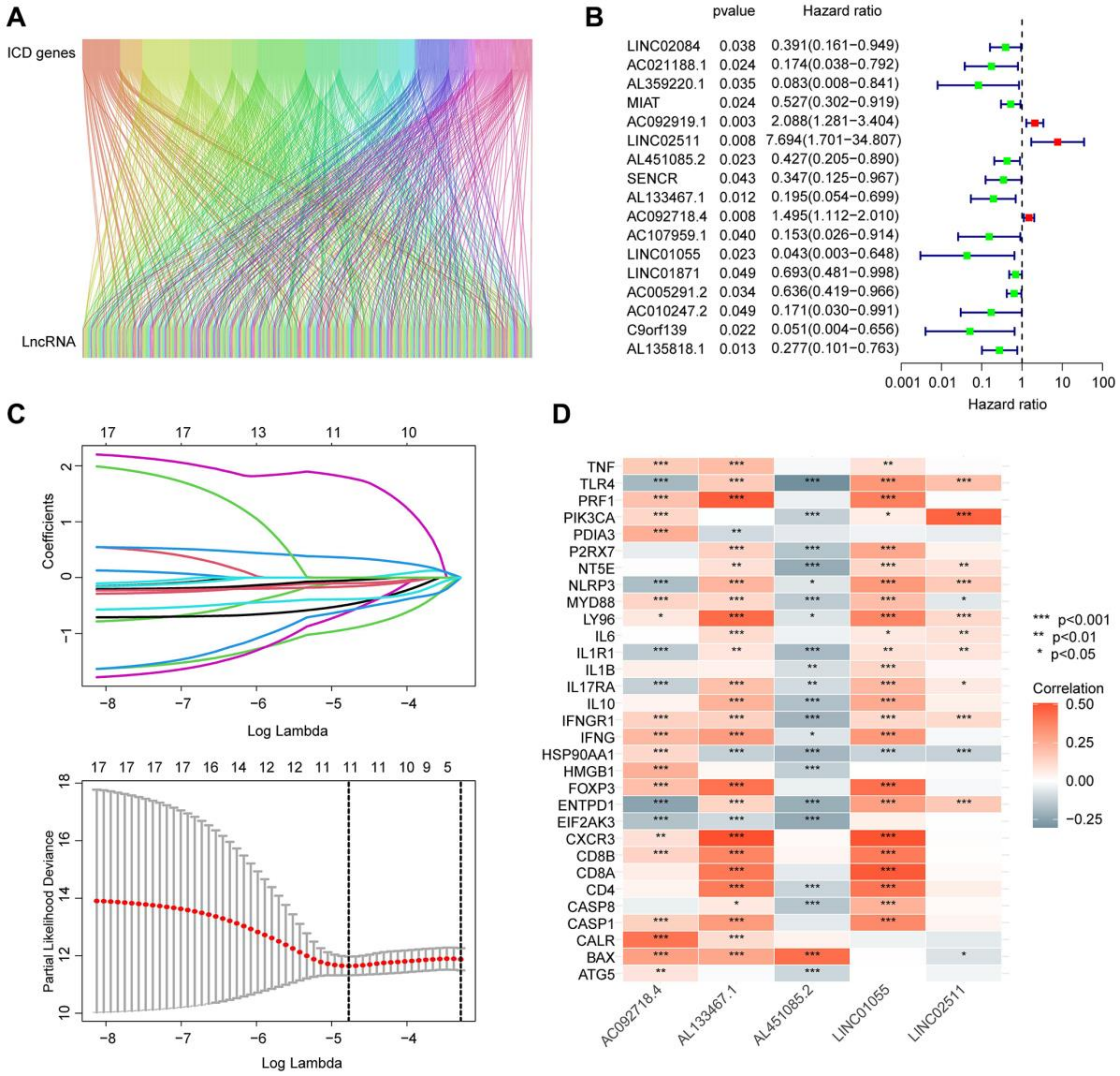
**Screening and identification of prognostic-associated ICDRLs.** (**A**) Sankey diagram displays the relationship between ICD genes and ICDRLs. (**B**) ICDRLs associated with BRCA prognosis are filtered and selected. (**C**) LASSO analysis shows the minimum lambda value of the characteristic variables. (**D**) The correlation heatmap reveals the associations between five independent prognostic factors and ICD genes.

In this study, we utilized the Pearson correlation algorithm and set the threshold for significance at |r| > 0.4 and *p* < 0.05. Based on this criterion, we identified and collected 184 ICDRLs for subsequent analysis. A Sankey diagram was employed to display the significant associations between ICD genes and ICDRLs (Figure 1A). Using univariate Cox analysis, we evaluated the potential prognostic value of the 184 ICDRLs in BRCA and found 17 ICDRLs that were significantly associated with prognosis, including three risk factors and 14 beneficial factors (Figure 1B). Through LASSO analysis, we further selected the characteristic variables of ICDRLs that were associated with BRCA prognosis, and by performing multivariate Cox analysis, we ultimately determined five independent characteristic variables that could predict BRCA prognosis (Figure 1C). The correlation results suggest that these five characteristics, including AC092718.4, AL133467.1, LINC01055, LINC02511, and AL451085.2, are significantly correlated with most ICD genes (Figure 1D). Specifically, AC092718.4, AL133467.1, LINC01055, and LINC02511 are positively correlated, while AL451085.2 is negatively correlated with most ICD genes.

### Constructing a prognostic risk model based on independent prognostic ICDRLs

**Figure 2:**
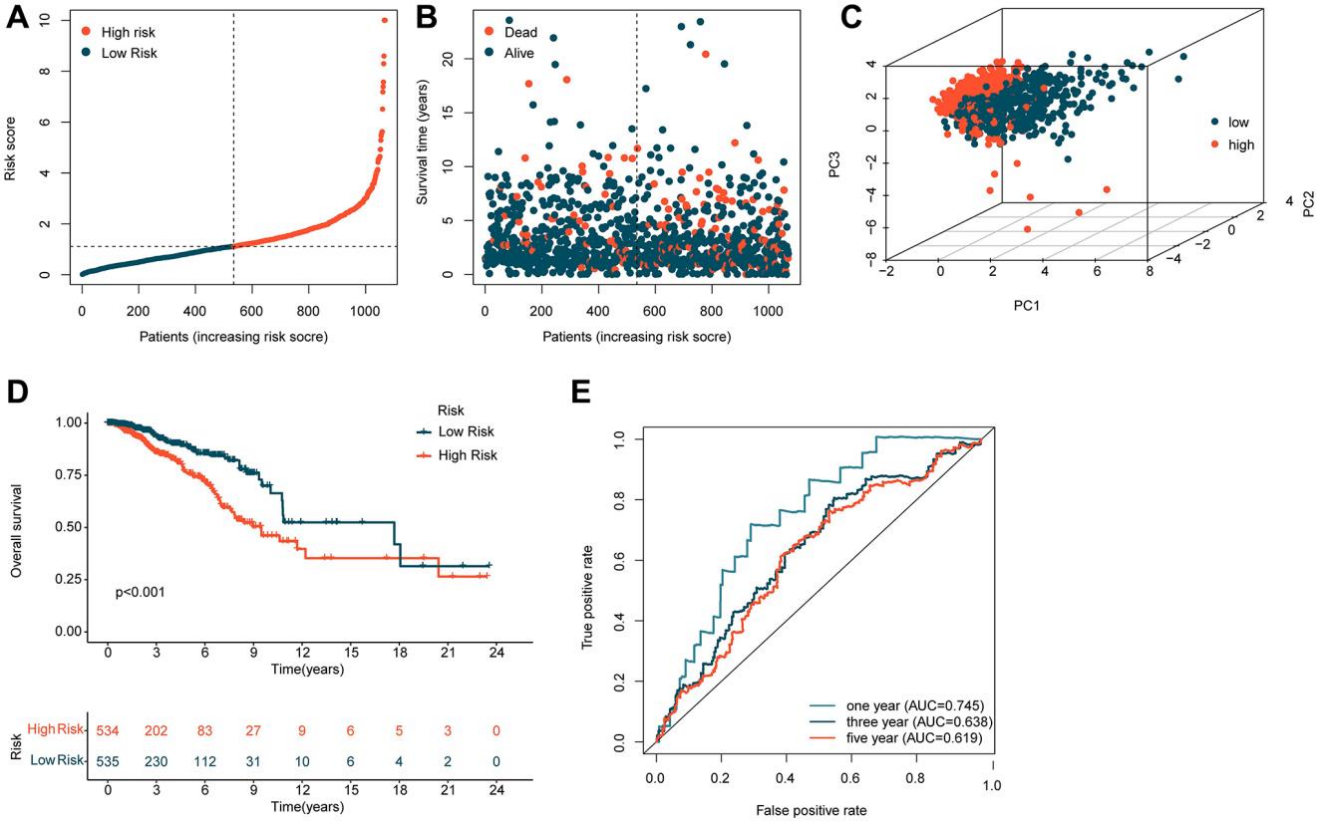
**Construction of a risk model based on five independent ICDRLs prognostic factors.** (**A**, **B**) Division of BRCA samples into two risk subgroups. (**C**) PCA analysis of high and low-risk subgroups. (**D**) Assessment of clinical survival outcomes for risk subgroups of BRCA. (**E**) Time-dependent ROC curve analysis.

Based on the coefficients obtained from the multivariate Cox analysis for five independent prognostic factors, we calculated the risk score for each BRCA sample. To explore the potential association between the risk score and BRCA prognosis, we divided the BRCA samples into two risk subgroups based on the median risk score and constructed a novel risk model, as shown in Figure 2A, 2B. The unsupervised PCA model based on the five independent prognostic factors clearly distinguished the risk subgroups of BRCA (Figure 2C). Clinical survival outcomes indicated that the low-risk subgroup had better survival prognosis than the high-risk subgroup of BRCA (Figure 2D). Furthermore, time-dependent ROC curve analysis showed that the AUC values for 1-, 3-, and 5-year survival were 0.745, 0.638, and 0.619, respectively (Figure 2E). These findings suggest that the risk model constructed based on the five independent ICDRLs prognostic factors may be associated with BRCA prognosis and can provide insights into different clinical survival outcomes.

### Evaluation of the independence of ICDRLs prognostic features

**Figure 3:**
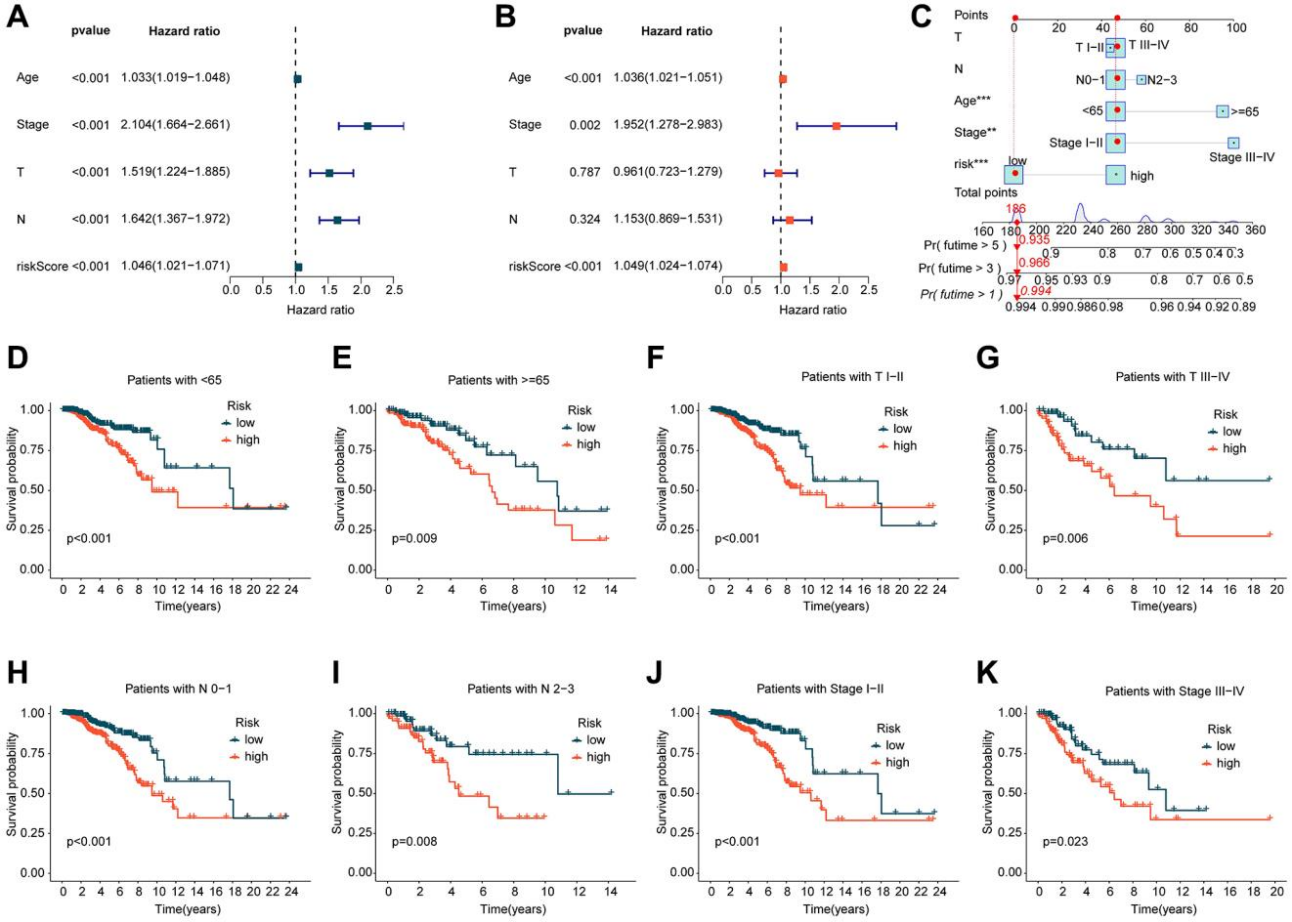
**Independent prognostic evaluation of ICDRLs prognostic features.** (**A**, **B**) Univariate and multivariate COX analysis calculating HR and *p*-values for different clinical-pathological variables and ICDRLs prognostic features. (**C**) Development of a nomogram based on different clinical-pathological variables and ICDRLs prognostic features. (**D**–**K**) Clinical survival outcome evaluation of risk subgroups based on ICDRLs prognostic features in different clinical-pathological features.

In view of the potential role of ICDRLs prognostic features in predicting the clinical survival outcome of BRCA, we integrated the clinical information of each BRCA sample and explored the independent prognostic value of each variable. We conducted both univariate and multivariate Cox analyses and calculated the hazard ratio (HR), *p*-value, and risk score for different clinical variables. Univariate Cox analysis revealed that age, stage, T, N, and risk score were closely associated with adverse prognostic outcomes in BRCA (HR > 1, *p* < 0.001). Multivariate Cox analysis suggested that the prognostic model based on five independent ICDRLs prognostic factors was an independent prognostic factor for BRCA (Figure 3A, 3B). Additionally, we developed a nomogram based on different clinical variables and ICDRLs prognostic features to accurately analyze the survival probability of BRCA at 1, 3, and 5 years (Figure 3C). In addition to evaluating the independent prognostic value of ICDRLs prognostic features, we also explored their prognostic value in different clinical-pathological features. Based on the median value of ICDRLs risk scores, BRCA samples with different clinical-pathological features were classified into high- and low-risk subgroups (Figure 3D–3K). Clinical survival outcome analysis revealed that in different clinical-pathological subgroups, the clinical prognosis of low-risk group BRCA samples was better than that of high-risk group samples. We therefore conclude that the prognostic model based on five ICDRLs features is an independent prognostic factor for BRCA and can accurately evaluate the clinical prognosis of BRCA in different clinical-pathological features.

### Validation of ICDRLs prognostic features in different independent cohorts

**Figure 4:**
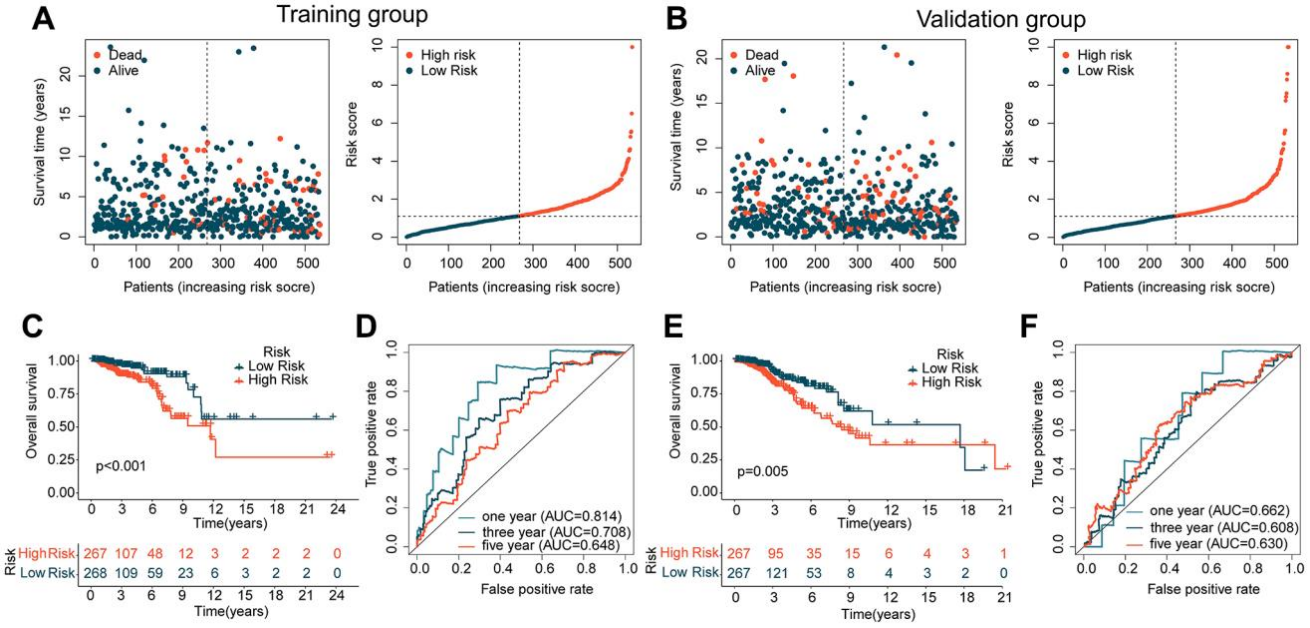
**Analysis of ICDRLs prognostic features in different independent cohorts.** (**A**, **B**) Risk subgroups based on ICDRLs prognostic features in the training and validation cohorts. (**C**) Clinical prognostic survival analysis in the training cohort. (**D**) Time-related ROC curve analysis in the training cohort. (**E**) Clinical prognostic survival analysis in the validation cohort. (**F**) Time-related ROC curve analysis in the validation cohort.

In the subsequent study, we further explored the independence and accuracy of ICDRLs prognostic features. Using the R script “caret” and a 1:1 random split, we divided BRCA samples from TCGA into training and validation cohorts. Based on the coefficients calculated by the multivariate Cox model and the expression of the five ICDRLs, we computed the risk score for each BRCA sample in the training and validation cohorts and classified them into high- and low-risk subgroups according to the median risk score (Figure 4A, 4B). In the training cohort, we found that the low-risk subgroup had a better clinical prognosis for BRCA (Figure 4C). Time-dependent ROC curves indicated AUC values of 0.814, 0.708, and 0.648 for 1-, 3-, and 5-year survival, respectively (Figure 4D). In the validation cohort, we observed consistent results with the training cohort, with the low-risk subgroup showing better clinical outcomes compared to the high-risk subgroup (Figure 4E). The time-dependent ROC curves showed AUC values of 0.662, 0.608, and 0.630 for 1-, 3-, and 5-year survival, respectively (Figure 4F). Based on the results of these two independent cohorts, we conclude that the prognostic features based on the five ICDRLs can accurately evaluate the prognosis of BRCA.

### Exploration of potential mechanisms based on the risk subgroups of ICDRLs

**Figure 5:**
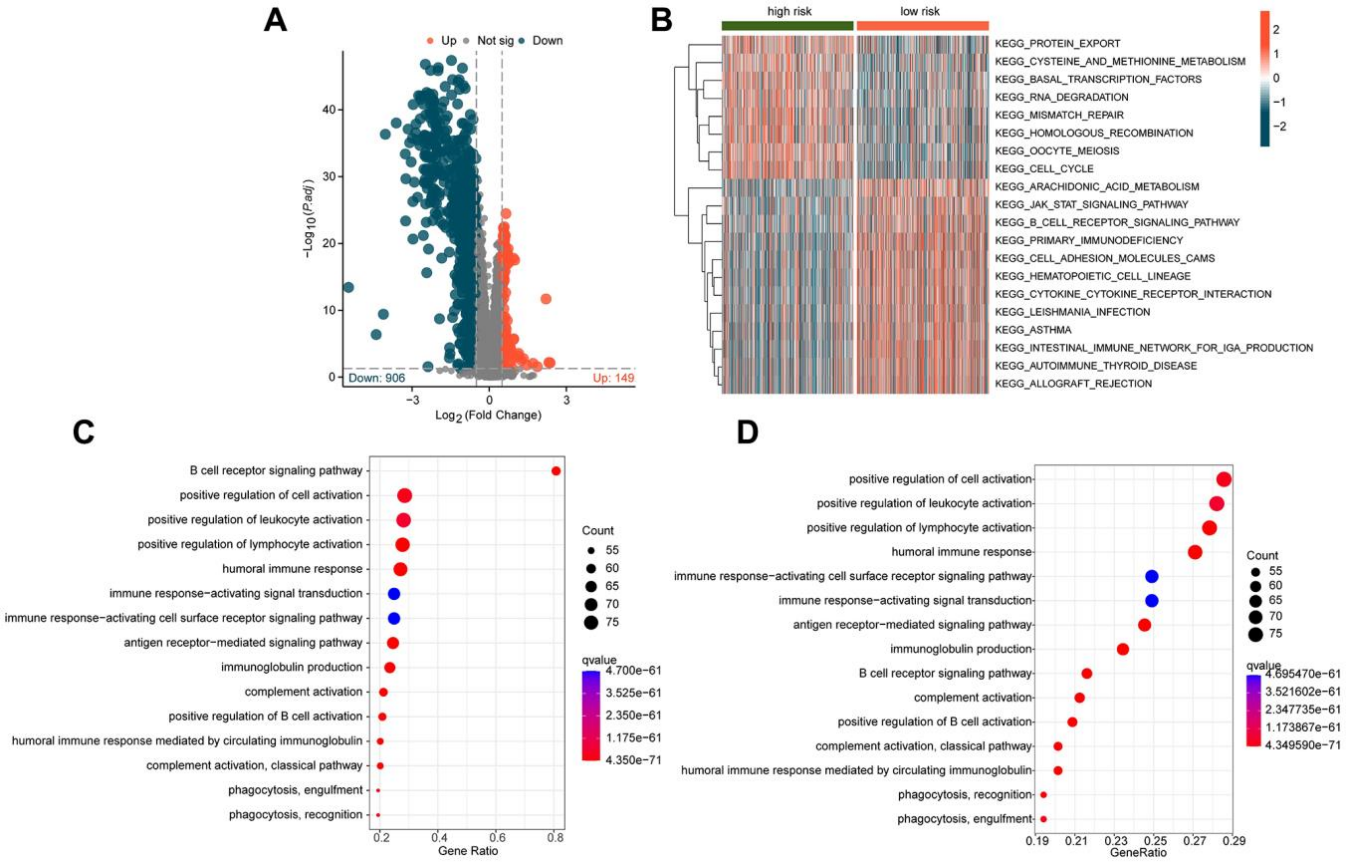
**Exploration of potential mechanisms in different ICDRLs risk subgroups.** (**A**) Differential gene expression analysis of risk subgroups. (**B**) GSVA analysis of different ICDRLs risk subgroups. (**C**, **D**) GO and KEGG enrichment analysis of differentially expressed genes.

To explore the potential regulatory mechanisms underlying the differential clinical survival outcomes between different ICDRLs risk subgroups, we analyzed some potential signaling pathways and molecular functional mechanisms between the subgroups. Based on the limma script, we investigated the differentially expressed genes (DEGs) between the two risk subgroups. As shown in Figure 5A, we obtained 149 significantly upregulated DEGs and 906 significantly downregulated DEGs according to the differential analysis. The GSVA heatmap revealed that some immune-related signaling pathways, such as B cell receptor signaling pathway and cytokine-cytokine receptor interaction were significantly upregulated in the low-risk subgroup, while some cancer-related signaling pathways, such as cell cycle, mismatch repair and RNA degradation were significantly upregulated in the high-risk subgroup (Figure 5B). Furthermore, we conducted GO and KEGG enrichment analyses based on these DEGs (Figure 5C, 5D). The GO results indicated significant enrichment of DEGs in immune functional signaling pathways such as B cell receptor signaling pathway, positive regulation of cell activation, and positive regulation of leukocyte activation, while the KEGG enrichment results suggested significant correlation between DEGs and a series of immune-related signaling pathways, involving positive regulation of lymphocyte activation, positive regulation of leukocyte activation, and positive regulation of cell activation. Based on these results, we hypothesize that the differentially expressed genes based on ICDRLs risk subgroups are related to immune functional signaling pathways, and the changes in immune signaling pathways may be the potential mechanism causing different clinical survival outcomes between risk subgroups.

### Assessment of immune infiltration in ICDRLs risk subgroups

**Figure 6:**
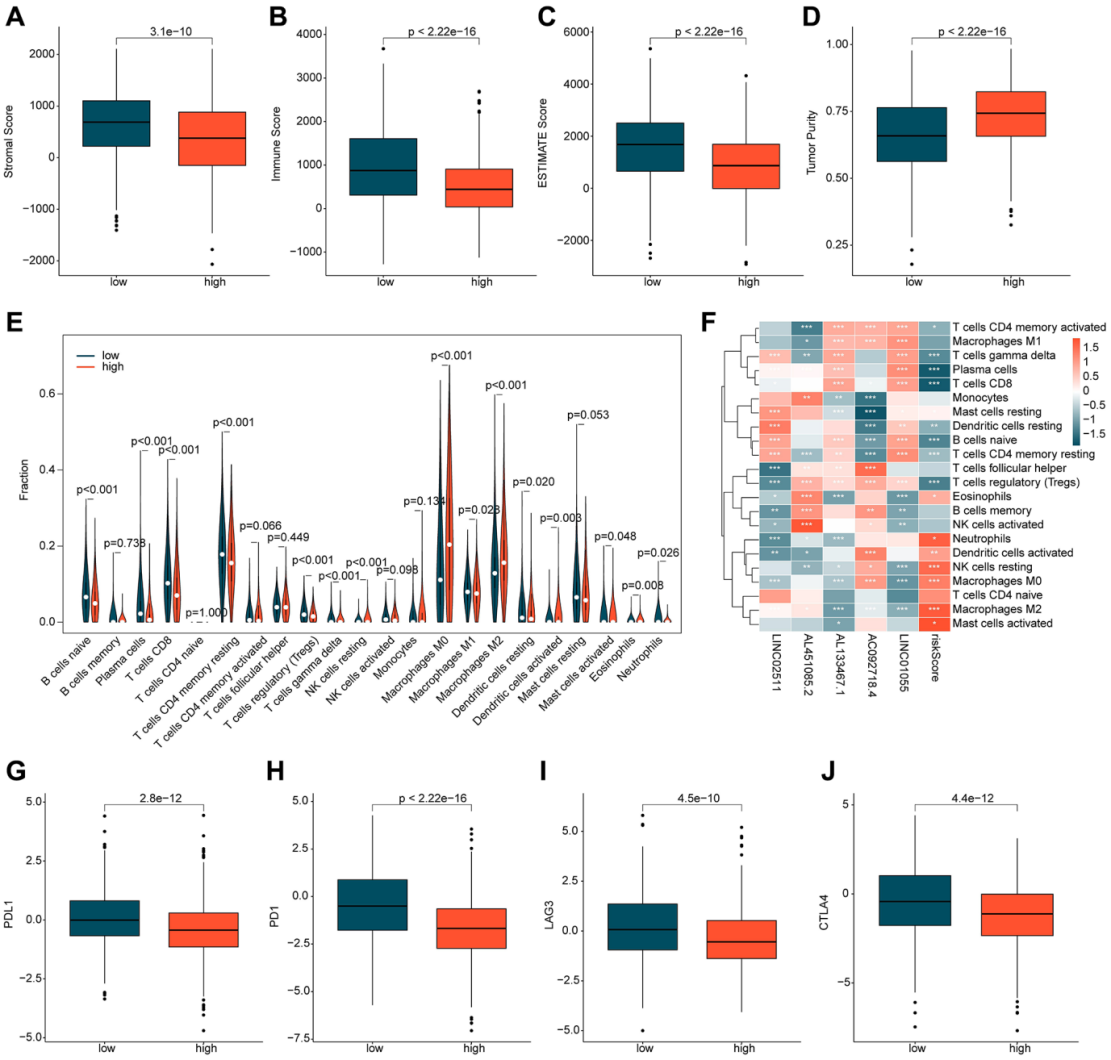
**Immune infiltration assessment of ICDRLs risk subgroups.** (**A**–**D**) Evaluation of immune status. (**E**) Evaluation of the proportions of 22 immune cells. (**F**) Analysis of the correlation between ICDRLs prognostic features, prognostic factors, and immune-infiltrating cells. (**G**–**J**) Analysis of the expression profiles of immune checkpoints in ICDRLs risk subgroups.

Based on the potential mechanisms of the ICDRLs risk subgroups, we speculate that immune-related changes may be the key factors that contribute to the differential outcomes observed in the ICDRLs risk subgroups. In light of this, we further explored the immune infiltration characteristics between different risk subgroups. Firstly, we employed the ESTIMATE algorithm to evaluate the immune status between two risk subgroups. The results indicate that the stromal, immune, and ESTIMATE scores were significantly lower in the high-risk group compared to the low-risk group, while the tumor purity was higher, suggesting a significant difference in immune status between the two risk subgroups (Figure 6A–6D). Using the CIBERSORT algorithm, we further assessed the immune infiltration characteristics of BRCA in the ICDRLs risk subgroups (Figure 6E). The proportion of 22 immune cells infiltrating the tumors suggests that the proportions of B cells naive, plasma cells, T cells CD8^+^, T cells CD4^+^ memory resting, T cells regulatory (Tregs), T cells gamma delta, macrophages M1, dendritic cells resting, mast cells activated, and neutrophils were higher in the low-risk group than in the high-risk group, while the proportions of NK cells resting, macrophages M0, macrophages M2, dendritic cells activated, and eosinophils were significantly lower in the high-risk group. Correlation analysis suggests that the ICDRLs risk score was significantly positively correlated with neutrophils, dendritic cells activated, NK cells resting, macrophages M0, eosinophils, mast cells activated, and mast cells resting, and significantly negatively correlated with T cells CD4 memory activated, T cells gamma delta, plasma cells, T cells CD8, mast cells resting, dendritic cells resting, B cells naive, and T cells CD4 memory resting. Meanwhile, we also observed a significant correlation between five prognostic factors and immune cell infiltration (Figure 6F). Immune checkpoint results indicate that the expression levels of PD-L1, PD-1, LAG3, and CTLA4 were significantly higher in the low-risk subgroup than in the high-risk subgroup (Figure 6G–6J). In summary, these results suggest significant differences in immune infiltration between different risk subgroups and may be related to the response to immune therapy.

### Analysis of immune therapy response and mutation burden characteristics

**Figure 7:**
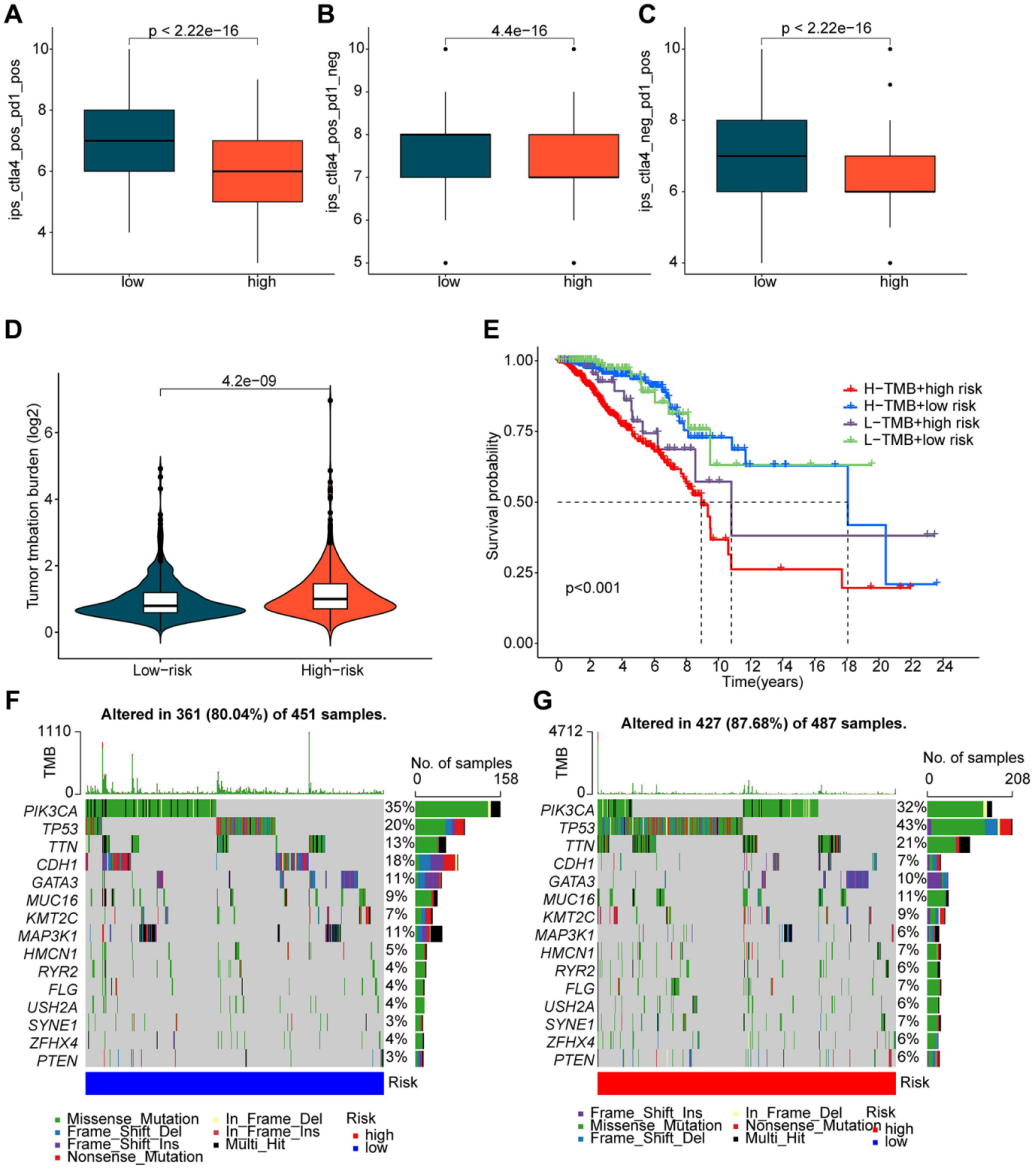
**Evaluation of immune therapy response and mutation load characteristics of ICDRLs risk subtypes.** (**A**–**C**) IPS scores predicting ICDRLs risk subtypes based on the TCIA database. (**D**) Tumor mutation load score of ICDRLs risk subtypes. (**E**) Clinical prognosis analysis of high and low mutation load groups based on ICDRLs risk subtypes. (**F**, **G**) Assessment of somatic mutation frequency in ICDRLs risk subtypes.

The application of immunotherapy strategies in BRCA treatment has shown remarkable potential. We conducted further evaluation of the immune therapy response of BRCA to PD-1 and CTLA4 in different ICDRLs risk subgroups. Utilizing the TCIA database, we predicted the immune therapy response of BRCA samples in both low and high-risk subgroups. The results demonstrated that BRCA samples in the low-risk group exhibited a significantly higher response to PD-1 and CTLA-4 therapy compared to the high-risk group (Figure 7A–7C). TMB analysis indicated that BRCA samples in the high-risk subgroup displayed a higher TMB score. Moreover, the clinical prognostic outcome of BRCA in the low-risk group was significantly better than that of the high-risk group in different mutational congruence subgroups (Figure 7D, 7E). The somatic mutation landscape results revealed that among 451 samples in the low-risk subgroup, 361 had somatic mutations, whereas among 487 samples in the high-risk subgroup, 427 had somatic mutations. In comparison to the mutation characteristics of the high-risk subgroup, we observed significantly lower mutation frequencies of TP53 and TTN in the low-risk subgroup. Notably, the mutation frequencies of PIK3CA, CDH1, and MAP3K1 were significantly lower in the high-risk subgroup than in the low-risk subgroup (Figure 7F, 7G). Based on these findings, we postulate that the ICDRLs prognostic characteristics based on five prognostic factors can reflect the immune therapy response of different risk subgroups and are associated with mutation feature.

### Prediction of chemotherapeutic drugs based on ICDRLs risk subtypes

**Figure 8:**
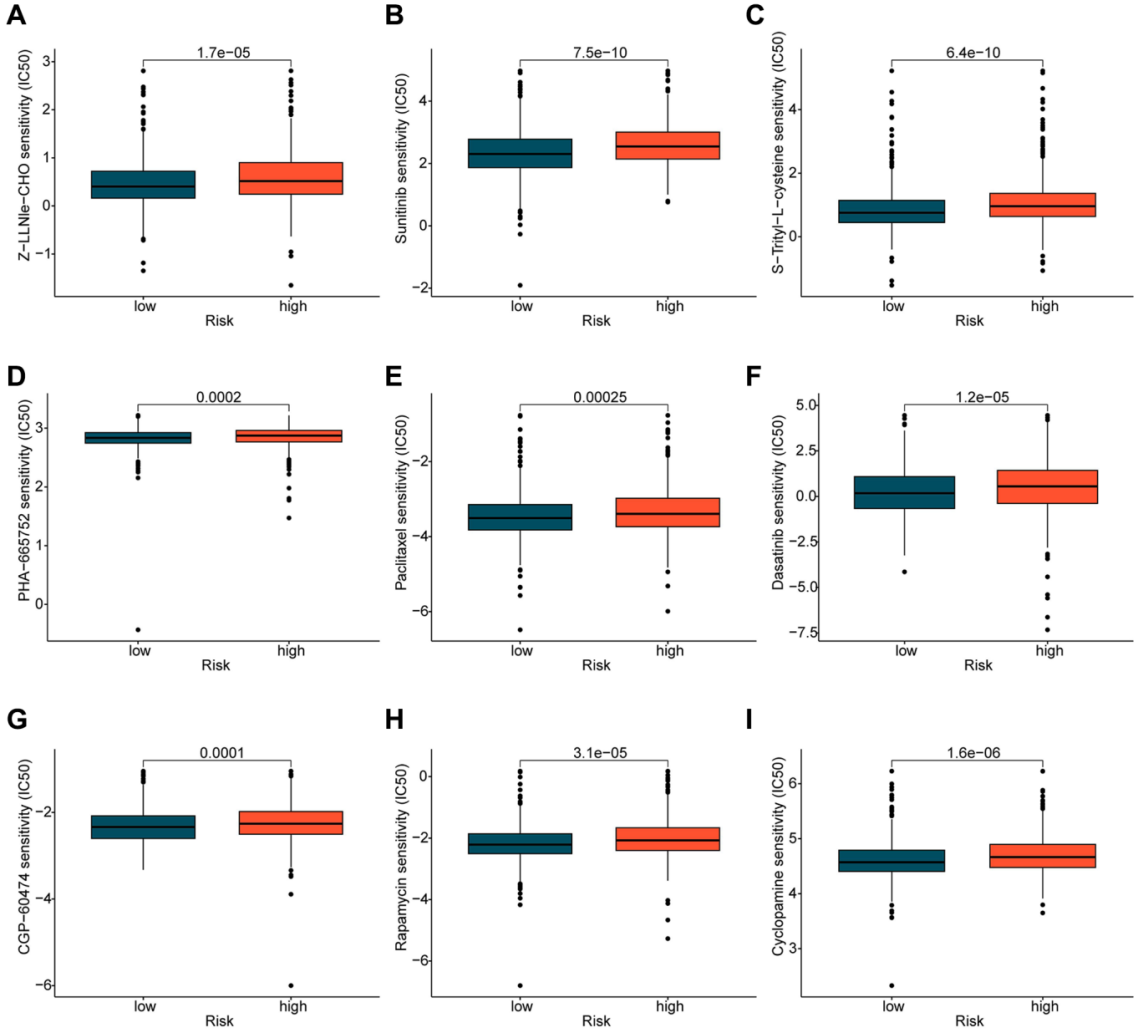
**Prediction of potential chemotherapy drugs.** Predicted IC50 values of (**A**) Z-LLNle-CHO, (**B**) Sunitinib, (**C**) S-Trityl-L-cysteine, (**D**) PHA-665752, (**E**) Paclitaxel, (**F**) Dasatinib, (**G**) CGP-60474, (**H**) Rapamycin, and (**I**) Cyclopamine in the ICDRLs risk subtypes.

In addition to immunotherapy, chemotherapeutic treatment represents a crucial therapeutic approach for patients diagnosed with BRCA. In further investigations, we expanded our research by predicting the potential benefit of certain chemotherapy drugs for BRCA treatment, using the GDSC database. As illustrated in Figure 8, we identified significantly higher IC50 values of Z-LLNle-CHO, Sunitinib, S-Trityl-L-cysteine, PHA-665752, Paclitaxel, Dasatinib, CGP-60474, Cyclopamine, and Rapamycin in the high-risk group as opposed to the low-risk group. This finding implies that individuals diagnosed with BRCA and classified in the high-risk group may display a greater degree of sensitivity towards these chemotherapy drugs.

### qRT-PCR validation of 5 prognostic ICDRLs

**Figure 9:**
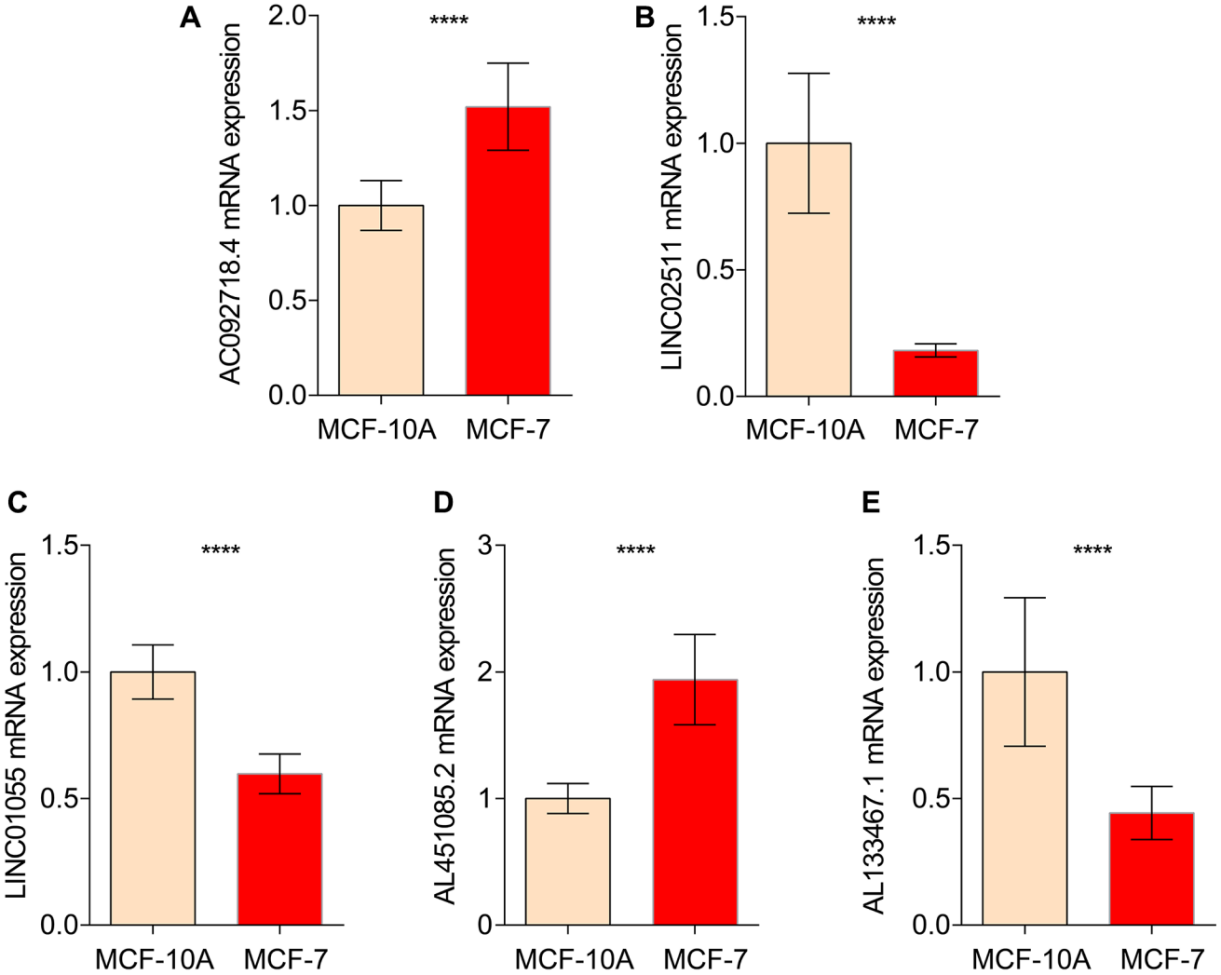
**qRT-PCR analysis of 5 ICDRLs in MCF-10A and MCF7.** mRNA expressions of AC092718.4 (**A**) LINC02511 (**B**) LINC01055 (**C**) AL451085.2 (**D**) AL133467.1 (**E**) in both MCF-10A and MCF7 cells.

In order to further verify the abnormal expressions of ICDRLs in BRCA, human normal mammary epithelial cell line MCF-10A and BRCA cell line MCF-7 were utilized. MCF-7 had higher mRNA expressions of AC092718.4, AL451085.2, while lower LINC02511, AL133467.1 and LINC01055 expressions in comparison to control cell line MCF-10A. All five ICDRLs showed abnormal expressions with significant differences *in vitro*, which was in line with the bioinformatics results (Figure 9).

### Over-expression of LINC01055 inhibits the migration and invasion of BRCA cells

**Figure 10:**
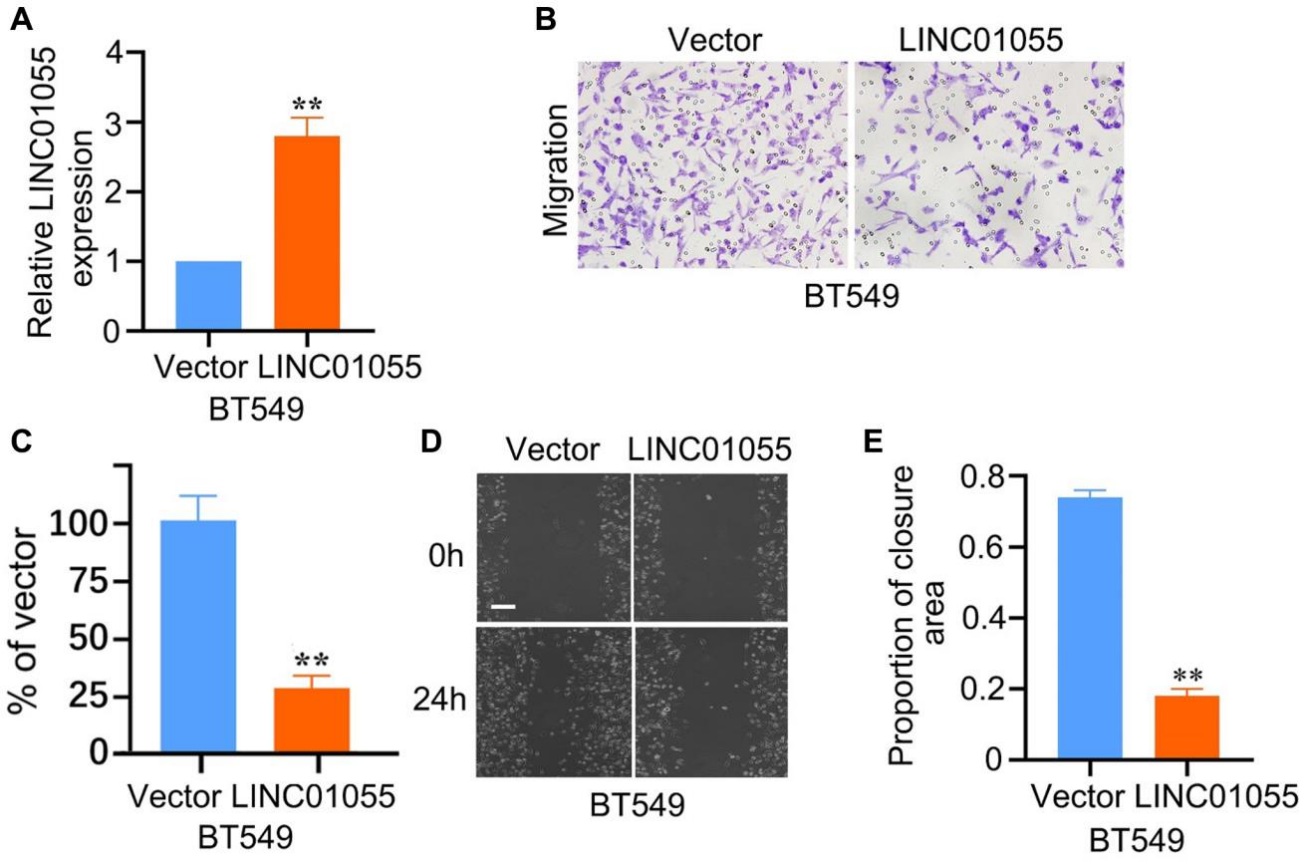
**Effects of overexpression of LINC01055 on migration and invasion ability of BRCA cells.** (**A**) Relative expression of LINC01055 detected by qRT-PCR in BT549 cells (*n* = 3). (**B**, **C**) Transwell analysis detected the effect of overexpressed LINC01055 on the invasion ability of BT549 (*n* = 3) (×100). (**D**, **E**) Scratch assay detected the migration ability of BT549 by 0 and 24 hours (*n* = 3) (×40).

To further verify whether LINC01055 affects the progression of BRCA, we transfected human BRCA cell line BT-549 with an adenovirus vector overexpressing LINC01055. qRT-PCR results showed that LINC01055 was significantly overexpressed compared to the negative control (Figure 10A). Scratch assay and Transwell analysis were subsequently performed to evaluate the role of LINC01055 in BRCA migration and invasion. Transwell results showed that overexpression of LINC01055 significantly reduced the number of BT-549 cells passing through the lower chamber in comparison to the control group (Figure 10B, 10C). Similarly, scratches in the vector control group continued to heal over time, while overexpression of LINC01055 inhibited the ability of cells to migrate to the scratched area (Figure 10D, 10E). These results suggest that overexpression of LINC01055 can inhibit the migration and invasion of BRCA cells.

## DISCUSSION

We established a prognostic model for BRCA based on five ICDRLs, and subsequently successfully confirmed the value of this model in guiding prognostic stratification. *In vitro* experiment also showed significant expression differences with the same trend in comparison to bioinformatics results.

Evidence has shown that ICDRLs can be used to characterize the infiltration of immune cells in tumors, which are potential targets for cancer therapy and have predictive value for survival and prognosis [19]. In addition, gene signatures developed by the combination of high-throughput sequencing technology and bioinformatics are widely used in individualized treatment and prognosis assessment, and their predictive accuracy is better than that of single biomarkers [20]. Therefore, it is necessary to establish ICDRLs features for the treatment and prognosis of BRCA patients. Among the 5 ICDRLs screened, AL133467.1, AL451085.2, and AC092718.4 were previously reported to be prognostic features of BRCA [21–23]. LINC01055 was used as a prognostic biomarker for colorectal cancer [24]. LINC02511 has not been publicly reported yet.

Tumor immune microenvironment (TME) plays an important role in the occurrence, development and prognosis of BRCA [25]. Some pathological subgroups of breast tumors are rich in tumor-infiltrating lymphocytes (TIL), which can be used as reliable prognostic biomarkers for BRCA and are associated with clinical responsiveness to chemotherapy agents such as docetaxel or doxorubicin. A 10% increase in TIL was associated with a varying reduction in the risk of recurrence and death [26–28]. In particular, infiltration of cytotoxic CD8^+^ T lymphocytes, CD4^+^ T lymphocytes, and tumor-associated macrophages (TAM) predicted favorable results [29]. Our results showed that the levels of CD8^+^ T cells and CD4^+^ T cells in the high-risk group were significantly lower than those in the low-risk group, suggesting that the high-risk group may have a lower immunosuppressive immune microenvironment of TIL, which may lead to a poorer prognosis in the high-risk group. Because chemotherapy agents can enhance antitumor immune responses by clearing immunosuppressive cells or inducing neoantigen release from tumor cells, higher levels of TIL can improve tumor response to immune checkpoint inhibitors [12]. We also found that the high-risk group had lower levels of TIL and a poorer response to immune checkpoint inhibitors. Among the differences in the components of immune infiltrating cells in different stratifications, we noticed that the high-risk group had lower levels of eosinophils in the immune microenvironment. Unforeseen roles for eosinophils have been found in a variety of environments that go well beyond allergic inflammation, Including carcinogenesis [30]. Eosinophils infiltrate multiple tumors and can regulate tumor progression either directly by interacting with tumor cells or indirectly by shaping the TME. Depending on the tumor type, eosinophils may exhibit pro-tumor or antitumor functions [30]. Activated eosinophils are essential for tumor rejection in the presence of tumor-specific CD8^+^ T cells. Tumor homing eosinophils secrete chemo-attractants that direct T cells into the tumor, leading to tumor eradication. At the same time, activated eosinophils trigger substantial changes in the tumor microenvironment, including macrophage polarization and normalization of tumor vasculature [31, 32]. In addition, the correlation between eosinophils’ response to cancer immunotherapy [30, 33] is of great significance for eosinophil in BRCA.

There are still limitations and weaknesses in our study. The bioinformatic results only got a preliminary *in vitro* validation, and their biological functions need to be further elucidated. Secondly, we described the correlation between ICD-related risk model and tumor-infiltrating immune components. However, due to the complexity, we did not further study the causal relationship between the two. Third, there may be some deviations in case inclusion and data processing in retrospective studies since only TCGA cohort was involved in study. Clinical sample collection and external validation will be implemented in the future.

## Supplementary Materials

Supplementary Tables 1 and 3

Supplementary Table 2

## References

[1] Sung H, Ferlay J, Siegel RL, Laversanne M, Soerjomataram I, Jemal A, Bray F. Global Cancer Statistics 2020: GLOBOCAN Estimates of Incidence and Mortality Worldwide for 36 Cancers in 185 Countries. CA Cancer J Clin. 2021; 71:209–49. 10.3322/caac.21660 33538338

[2] Woolston C. Breast cancer. Nature. 2015; 527:S101. 10.1038/527S101a 26580154

[3] Steeg PS. Tumor metastasis: mechanistic insights and clinical challenges. Nat Med. 2006; 12:895–904. 10.1038/nm1469 16892035

[4] Smyth LM, Piha-Paul SA, Won HH, Schram AM, Saura C, Loi S, Lu J, Shapiro GI, Juric D, Mayer IA, Arteaga CL, de la Fuente MI, Brufksy AM, et al. Efficacy and Determinants of Response to HER Kinase Inhibition in HER2-Mutant Metastatic Breast Cancer. Cancer Discov. 2020; 10:198–213. 10.1158/2159-8290.CD-19-0966 31806627 PMC7007377

[5] André F, Ciruelos E, Rubovszky G, Campone M, Loibl S, Rugo HS, Iwata H, Conte P, Mayer IA, Kaufman B, Yamashita T, Lu YS, Inoue K, et al, and SOLAR-1 Study Group. Alpelisib for PIK3CA-Mutated, Hormone Receptor-Positive Advanced Breast Cancer. N Engl J Med. 2019; 380:1929–40. 10.1056/NEJMoa1813904 31091374

[6] Schmid P, Adams S, Rugo HS, Schneeweiss A, Barrios CH, Iwata H, Diéras V, Hegg R, Im SA, Shaw Wright G, Henschel V, Molinero L, Chui SY, et al, and IMpassion130 Trial Investigators. Atezolizumab and Nab-Paclitaxel in Advanced Triple-Negative Breast Cancer. N Engl J Med. 2018; 379:2108–21. 10.1056/NEJMoa1809615 30345906

[7] Galluzzi L, Vitale I, Warren S, Adjemian S, Agostinis P, Martinez AB, Chan TA, Coukos G, Demaria S, Deutsch E, Draganov D, Edelson RL, Formenti SC, et al. Consensus guidelines for the definition, detection and interpretation of immunogenic cell death. J Immunother Cancer. 2020; 8:e000337. 10.1136/jitc-2019-000337 32209603 PMC7064135

[8] Deng H, Yang W, Zhou Z, Tian R, Lin L, Ma Y, Song J, Chen X. Targeted scavenging of extracellular ROS relieves suppressive immunogenic cell death. Nat Commun. 2020; 11:4951. 10.1038/s41467-020-18745-6 33009382 PMC7532538

[9] Alzeibak R, Mishchenko TA, Shilyagina NY, Balalaeva IV, Vedunova MV, Krysko DV. Targeting immunogenic cancer cell death by photodynamic therapy: past, present and future. J Immunother Cancer. 2021; 9:e001926. 10.1136/jitc-2020-001926 33431631 PMC7802670

[10] Xu M, Lu JH, Zhong YZ, Jiang J, Shen YZ, Su JY, Lin SY. Immunogenic Cell Death-Relevant Damage-Associated Molecular Patterns and Sensing Receptors in Triple-Negative Breast Cancer Molecular Subtypes and Implications for Immunotherapy. Front Oncol. 2022; 12:870914. 10.3389/fonc.2022.870914 35444934 PMC9013947

[11] Heimes AS, Schmidt M. Atezolizumab for the treatment of triple-negative breast cancer. Expert Opin Investig Drugs. 2019; 28:1–5. 10.1080/13543784.2019.1552255 30474425

[12] Keenan TE, Tolaney SM. Role of Immunotherapy in Triple-Negative Breast Cancer. J Natl Compr Canc Netw. 2020; 18:479–89. 10.6004/jnccn.2020.7554 32259782

[13] Pilones KA, Hensler M, Daviaud C, Kraynak J, Fucikova J, Galluzzi L, Demaria S, Formenti SC. Converging focal radiation and immunotherapy in a preclinical model of triple negative breast cancer: contribution of VISTA blockade. Oncoimmunology. 2020; 9:1830524. 10.1080/2162402X.2020.1830524 33150045 PMC7583495

[14] Voorwerk L, Slagter M, Horlings HM, Sikorska K, van de Vijver KK, de Maaker M, Nederlof I, Kluin RJC, Warren S, Ong S, Wiersma TG, Russell NS, Lalezari F, et al. Immune induction strategies in metastatic triple-negative breast cancer to enhance the sensitivity to PD-1 blockade: the TONIC trial. Nat Med. 2019; 25:920–8. 10.1038/s41591-019-0432-4 31086347

[15] Qiu X, Qu Y, Guo B, Zheng H, Meng F, Zhong Z. Micellar paclitaxel boosts ICD and chemo-immunotherapy of metastatic triple negative breast cancer. J Control Release. 2022; 341:498–510. 10.1016/j.jconrel.2021.12.002 34883139

[16] Wang Z, Chen J, Hu J, Zhang H, Xu F, He W, Wang X, Li M, Lu W, Zeng G, Zhou P, Huang P, Chen S, et al. cGAS/STING axis mediates a topoisomerase II inhibitor-induced tumor immunogenicity. J Clin Invest. 2019; 129:4850–62. 10.1172/JCI127471 31408442 PMC6819145

[17] Li Y, Zhang H, Li Q, Zou P, Huang X, Wu C, Tan L. CDK12/13 inhibition induces immunogenic cell death and enhances anti-PD-1 anticancer activity in breast cancer. Cancer Lett. 2020; 495:12–21. 10.1016/j.canlet.2020.09.011 32941949

[18] Qing X, Xu W, Liu S, Chen Z, Ye C, Zhang Y. Molecular Characteristics, Clinical Significance, and Cancer Immune Interactions of Angiogenesis-Associated Genes in Gastric Cancer. Front Immunol. 2022; 13:843077. 10.3389/fimmu.2022.843077 35273618 PMC8901990

[19] Denaro N, Merlano MC, Lo Nigro C. Long noncoding RNAs as regulators of cancer immunity. Mol Oncol. 2019; 13:61–73. 10.1002/1878-0261.12413 30499165 PMC6322193

[20] Yu Y, Feng X, Cang S. A two-microRNA signature as a diagnostic and prognostic marker of pancreatic adenocarcinoma. Cancer Manag Res. 2018; 10:1507–15. 10.2147/CMAR.S158712 29942152 PMC6005310

[21] Wang Y, Liang X, Zhou Z, Hou Z, Yang J, Gao Y, Yang C, Chen T, Li C. Prevalence and Numbers of Diabetes Patients with Elevated BMI in China: Evidence from a Nationally Representative Cross-Sectional Study. Int J Environ Res Public Health. 2022; 19:2989. 10.3390/ijerph19052989 35270682 PMC8910421

[22] Tao S, Tao K, Cai X. Necroptosis-Associated lncRNA Prognostic Model and Clustering Analysis: Prognosis Prediction and Tumor-Infiltrating Lymphocytes in Breast Cancer. J Oncol. 2022; 2022:7099930. 10.1155/2022/7099930 35528236 PMC9068297

[23] Shi GJ, Zhou Q, Zhu Q, Wang L, Jiang GQ. A novel prognostic model associated with the overall survival in patients with breast cancer based on lipid metabolism-related long noncoding RNAs. J Clin Lab Anal. 2022; 36:e24384. 10.1002/jcla.24384 35441740 PMC9169174

[24] Qin F, Xu H, Wei G, Ji Y, Yu J, Hu C, Yuan C, Ma Y, Qian J, Li L, Huo J. A Prognostic Model Based on the Immune-Related lncRNAs in Colorectal Cancer. Front Genet. 2021; 12:658736. 10.3389/fgene.2021.658736 33959151 PMC8093825

[25] Shah L, Latif A, Williams KJ, Tirella A. Role of stiffness and physico-chemical properties of tumour microenvironment on breast cancer cell stemness. Acta Biomater. 2022; 152:273–89. 10.1016/j.actbio.2022.08.074 36087866

[26] Adams S, Gray RJ, Demaria S, Goldstein L, Perez EA, Shulman LN, Martino S, Wang M, Jones VE, Saphner TJ, Wolff AC, Wood WC, Davidson NE, et al. Prognostic value of tumor-infiltrating lymphocytes in triple-negative breast cancers from two phase III randomized adjuvant breast cancer trials: ECOG 2197 and ECOG 1199. J Clin Oncol. 2014; 32:2959–66. 10.1200/JCO.2013.55.0491 25071121 PMC4162494

[27] Loi S, Michiels S, Salgado R, Sirtaine N, Jose V, Fumagalli D, Kellokumpu-Lehtinen PL, Bono P, Kataja V, Desmedt C, Piccart MJ, Loibl S, Denkert C, et al. Tumor infiltrating lymphocytes are prognostic in triple negative breast cancer and predictive for trastuzumab benefit in early breast cancer: results from the FinHER trial. Ann Oncol. 2014; 25:1544–50. 10.1093/annonc/mdu112 24608200

[28] Loi S, Sirtaine N, Piette F, Salgado R, Viale G, Van Eenoo F, Rouas G, Francis P, Crown JP, Hitre E, de Azambuja E, Quinaux E, Di Leo A, et al. Prognostic and predictive value of tumor-infiltrating lymphocytes in a phase III randomized adjuvant breast cancer trial in node-positive breast cancer comparing the addition of docetaxel to doxorubicin with doxorubicin-based chemotherapy: BIG 02-98. J Clin Oncol. 2013; 31:860–7. 10.1200/JCO.2011.41.0902 23341518

[29] Matsumoto H, Thike AA, Li H, Yeong J, Koo SL, Dent RA, Tan PH, Iqbal J. Increased CD4 and CD8-positive T cell infiltrate signifies good prognosis in a subset of triple-negative breast cancer. Breast Cancer Res Treat. 2016; 156:237–47. 10.1007/s10549-016-3743-x 26960711

[30] Grisaru-Tal S, Itan M, Klion AD, Munitz A. A new dawn for eosinophils in the tumour microenvironment. Nat Rev Cancer. 2020; 20:594–607. 10.1038/s41568-020-0283-9 32678342

[31] Hollande C, Boussier J, Ziai J, Nozawa T, Bondet V, Phung W, Lu B, Duffy D, Paradis V, Mallet V, Eberl G, Sandoval W, Schartner JM, et al. Inhibition of the dipeptidyl peptidase DPP4 (CD26) reveals IL-33-dependent eosinophil-mediated control of tumor growth. Nat Immunol. 2019; 20:257–64. 10.1038/s41590-019-0321-5 30778250

[32] Carretero R, Sektioglu IM, Garbi N, Salgado OC, Beckhove P, Hämmerling GJ. Eosinophils orchestrate cancer rejection by normalizing tumor vessels and enhancing infiltration of CD8(+) T cells. Nat Immunol. 2015; 16:609–17. 10.1038/ni.3159 25915731

[33] Ghebeh H, Elshenawy MA, AlSayed AD, Al-Tweigeri T. Peripheral blood eosinophil count is associated with response to chemoimmunotherapy in metastatic triple-negative breast cancer. Immunotherapy. 2022; 14:189–99. 10.2217/imt-2021-0149 34984928

