# Supplementary material for: Comprehensive analysis of ICD-related lncRNAs in predicting risk stratification, clinical prognosis and immune response for breast cancer
Source: Aging (Albany NY). 2023 Sep 9;15(17):8833–50. doi: 10.18632/aging.205002 (PMC10522379; doi:10.18632/aging.205002)
Supplement: Supplementary Tables 1 and 3 [file aging-15-205002-s001.pdf]

SUPPLEMENTARY TABLES

Supplementary Table 1. The 33 ICD-related genes.

Gene
IL17RA
IL1R1
PIK3CA
CD4
IFNG
PRF1
CXCR3
CD8A
CD8B
P2RX7
NLRP3
IL10
TLR4
ENTPD1
ATG5
IFNB1
IL6
EIF2AK3
IL17A
LY96
FOXP3
HMGB1
HSP90AA1
BAX
PDIA3
CALR
CASP8
MYD88
IFNGR1
CASP1
IL1B
TNF
NT5E

Supplementary Table 3. The primer sequences of the lncRNAs.

	**F**	**R**
LINC02511	CAAGCAATGGATGTCGGAGC	AGGTCTTGCCCAGACAGGTA
AL451085.2	AGACGCTACGCCTGAAAACA	CTAGCGGAGACGACCCTTTC
AL133467.1	AGACGCTACGCCTGAAAACA	CTAGCGGAGACGACCCTTTC
AC092718.4	GCCTCTGGATCAGATGAGCG	GCCAGTTACTAACCCCGCAT
LINC01055	CGTGAGTTGATTGGACCCCA	GCTCTGCACTGGTTTGTTGG
